# Host-response-based gene signatures for tuberculosis diagnosis: A systematic comparison of 16 signatures

**DOI:** 10.1371/journal.pmed.1002786

**Published:** 2019-04-23

**Authors:** Hayley Warsinske, Rohit Vashisht, Purvesh Khatri

**Affiliations:** 1 Institute for Immunity, Transplantation and Infection, Stanford University, Stanford, California, United States of America; 2 Center for Biomedical Informatics, Department of Medicine, Stanford University, Stanford, California, United States of America; John Hopkins University, UNITED STATES

## Abstract

**Background:**

The World Health Organization (WHO) and Foundation for Innovative New Diagnostics (FIND) have published target product profiles (TPPs) calling for non-sputum-based diagnostic tests for the diagnosis of active tuberculosis (ATB) disease and for predicting the progression from latent tuberculosis infection (LTBI) to ATB. A large number of host-derived blood-based gene-expression biomarkers for diagnosis of patients with ATB have been proposed to date, but none have been implemented in clinical settings. The focus of this study is to directly compare published gene signatures for diagnosis of patients with ATB across a large, diverse list of publicly available gene expression datasets, and evaluate their performance against the WHO/FIND TPPs.

**Methods and findings:**

We searched PubMed, Gene Expression Omnibus (GEO), and ArrayExpress in June 2018. We included all studies irrespective of study design and enrollment criteria. We found 16 gene signatures for the diagnosis of ATB compared to other clinical conditions in PubMed. For each signature, we implemented a classification model as described in the corresponding original publication of the signature. We identified 24 datasets containing 3,083 transcriptome profiles from whole blood or peripheral blood mononuclear cell samples of healthy controls or patients with ATB, LTBI, or other diseases from 14 countries in GEO. Using these datasets, we calculated weighted mean area under the receiver operating characteristic curve (AUROC), specificity at 90% sensitivity, and negative predictive value (NPV) for each gene signature across all datasets. We also compared the diagnostic odds ratio (DOR), heterogeneity in DOR, and false positive rate (FPR) for each signature using bivariate meta-analysis. Across 9 datasets of patients with culture-confirmed diagnosis of ATB, 11 signatures had weighted mean AUROC > 0.8, and 2 signatures had weighted mean AUROC ≤ 0.6. All but 2 signatures had high NPV (>98% at 2% prevalence). Two gene signatures achieved the minimal WHO TPP for a non-sputum-based triage test. When including datasets with clinical diagnosis of ATB, there was minimal reduction in the weighted mean AUROC and specificity of all but 3 signatures compared to when using only culture-confirmed ATB data. Only 4 signatures had homogeneous DOR and lower FPR when datasets with clinical diagnosis of ATB were included; other signatures either had heterogeneous DOR or higher FPR or both. Finally, 7 of 16 gene signatures predicted progression from LTBI to ATB 6 months prior to sputum conversion with positive predictive value > 6% at 2% prevalence. Our analyses may have under- or overestimated the performance of certain ATB diagnostic signatures because our implementation may be different from the published models for those signatures. We re-implemented published models because the exact models were not publicly available.

**Conclusions:**

We found that host-response-based diagnostics could accurately identify patients with ATB and predict individuals with high risk of progression from LTBI to ATB prior to sputum conversion. We found that a higher number of genes in a signature did not increase the accuracy of the signature. Overall, the Sweeney3 signature performed robustly across all comparisons. Our results provide strong evidence for the potential of host-response-based diagnostics in achieving the WHO goal of ending tuberculosis by 2035, and host-response-based diagnostics should be pursued for clinical implementation.

## Abstract 2

Using 24 published datasets, Purvesh Khatri and colleagues compare the performance 16 gene signatures for diagnosis of patients with active tuberculosis.

## Author summary

**Why was this study done?:**

- There is an urgent need for a non-sputum-based triage test for diagnosis of active tuberculosis (ATB).
- The World Health Organization (WHO) has specified criteria for such a test that could be used to end tuberculosis by 2035.
- Several gene signatures measuring host immune response to *Mycobacterium tuberculosis* in blood samples have been proposed, but none has translated in clinical practice.

**What did the researchers do and find?:**

- Researchers compared 16 such gene signatures to investigate whether 1 or more of them could identify patients with ATB with the desired accuracy.
- The analysis found that 2 of the proposed gene signatures satisfied the WHO criteria for a non-sputum-based triage test across heterogeneous culture-confirmed datasets.
- Importantly, only 1 of these 2 signatures had a low false positive rate and no heterogeneity in its diagnostic accuracy, suggesting that it is generalizable across diverse patient populations.

**What do these findings mean?:**

- The findings strongly suggest that host-response-based diagnostics for ATB have the potential to aid in achieving the WHO goal of ending TB by 2035 and should be considered for clinical implementation.

## Introduction

The World Health Organization (WHO) has identified the need for a non-sputum-based triage test to rule out active tuberculosis (ATB) disease [1]. The WHO consensus meeting report describes that such a triage test should have 90% sensitivity and 70% specificity at minimum to end tuberculosis (TB) by 2035 [1]. In clinical practice, a triage test to rule out ATB requires high negative predictive value (NPV). WHO has also described the need for a test to predict progression from latent TB infection (LTBI) to ATB with >75% specificity and >75% sensitivity [2]. Further, the Foundation for Innovative New Diagnostics (FIND) and the New Diagnostics Working Group of the Stop TB Partnership have proposed a need for a prognostic test for TB risk that requires a positive predictive value (PPV) > 5.8% at a 2-year cumulative incidence of ATB of 2% (http://www.finddx.org/wp-content/uploads/2016/05/TPP-LTBIprogression.pdf).

Sputum culture is considered the gold standard for ATB diagnosis but takes 6–7 days for a positive diagnosis and up to 42 days for a confirmed negative diagnosis. Current sputum-based tests in clinical practice (e.g., smear microscopy, culture, and PCR-based assays) do not meet the desired target product profiles (TPPs), lack the sensitivity to reliably distinguish ATB from LTBI, and are prone to producing false negative results because sufficient bacilli-containing sputum samples can be difficult to obtain, especially from children and from individuals co-infected with HIV [3–12]. Sputum-bacilli tests cannot be used to identify patients with a high risk of progression because diagnosis with ATB is defined by the presence of bacilli in sputum [12]. A stool-based diagnostic test for ATB in children was shown to have 31.9% sensitivity at 99.7% specificity. These performance statistics are well suited for a diagnostic test for ruling in a patient with ATB, but not for a triage test to rule out ATB or for a progression test [13].

Recently, diagnostic gene signatures based on host immune response have been repeatedly demonstrated to accurately distinguish infection from other non-pathogenic inflammatory conditions [14], and to distinguish bacterial and viral infections [15,16]. Particularly for ATB, several host-response-based gene signatures have been proposed over the last decade for distinguishing patients with ATB from healthy controls and patients with LTBI and other diseases (ODs), and to predict progression from LTBI to ATB [17–29]. Collectively, these studies profiled whole blood or peripheral blood mononuclear cells (PBMCs) from samples that span a broad range of clinical conditions, including different age groups (children, adolescents, and adults), infection types (LTBI and ATB), and control (noninfectious) conditions. The number of genes in these signatures varies dramatically [17,20,23]. Notably, a variety of computational techniques—including support vector machine, random forest, linear discriminant analysis, logistic regression, and difference of means—have been applied to identify these signatures.

Despite extensive efforts, none of these TB gene signatures has been translated into a point of care (POC) diagnostic for several reasons. First, none of these signatures except 1 has been validated in prospective independent cohorts. As more gene signatures have been described, there has been a dearth of studies comparing these signatures with each other to verify if host-response-based signatures are appropriate for translation to clinical practice. Second, a majority of these gene signatures are composed of a large number of genes. Although a higher number of genes in a signature tends to increase accuracy [24], implementation of such a signature as a simple and cost-effective POC test is very difficult using current technology for measuring gene expression. Virtually all commercially available platforms for measuring transcriptional host response are limited by the number of genes they can measure. For instance, the Cepheid GeneXpert system, arguably the most widely used platform in TB diagnostics, can measure only up to 10 genes. Also, 1 or 2 of these genes need to be control genes, which further reduces the number of genes that can be used in a diagnostic signature to 8 or 9. Third, and most importantly, the generalizability of these transcriptional signatures to real-world patient populations in various clinical contexts is questionable. For instance, a 16-gene signature for predicting progression from LTBI to ATB developed using a single cohort from a single country was shown to lack generalizability to cohorts from other countries on the same continent [29]. In contrast, a 3-gene signature developed using heterogeneous cohorts from multiple countries has been shown to be more generalizable to other countries in retrospective [17] and prospective validation [11,30]. Another factor with substantial impact on the generalizability of these signatures is that the different statistical models used for creating these signatures are difficult to generalize across different populations and different measurement technologies. For instance, models based on *K*-nearest-neighbors clustering are difficult to generalize due to high sensitivity to batch effects and scaling within data [31].

The proliferation of host-response-based gene signatures despite the challenges described above raises several questions. First, do these signatures perform similarly to each other in different clinical contexts in different patient populations? If yes, the second question is, do 1 or more of these signatures have the potential to move towards translation into clinical practice cost-effectively? Third, an overarching question is, does host response to *Mycobacterium tuberculosis (Mtb)* have the potential to achieve the generalizability required to be used as a non-sputum-based triage test that meets the TPPs described by the WHO and other groups for ending TB by 2035?

We sought to answer these questions through a systematic comprehensive analysis of gene signatures for diagnosis of TB. To estimate the diagnostic accuracy of each of the published signatures, we reconstructed the classification model associated with each gene signature using the same discovery cohort as the original publication to the best of our ability. We then evaluated the accuracy of each signature in distinguishing patients with ATB from those with LTBI or ODs and healthy controls using publicly available gene expression datasets. We also evaluated whether these signatures could predict progression from LTBI to ATB prior to sputum conversion. We used specificity, sensitivity, comparison with various TPPs for ending TB by 2035, weighted mean area under the receiver operating characteristic curve (AUROC), PPV, NPV, false positive rate (FPR), heterogeneity across datasets, and diagnostic odds ratio (DOR) to evaluate the accuracy of various gene signatures.

## Methods

### Prospective analysis plan

We did not have a prospective analysis plan. Our goal from the beginning was broadly divided into the following steps that were modeled after a similar benchmarking analysis of biomarkers for sepsis [32]:

**Step 1:** Identify a set of gene signatures for diagnosis of ATB from literature. We define a “gene signature” as a set of genes derived from an analysis of whole transcriptome profiles that distinguishes patients with ATB from healthy controls or patients with LTBI or ODs.

**Step 2:** Identify a set of appropriate transcriptome datasets from PBMC or whole blood samples. A “transcriptome dataset” is a collection of “transcriptome profiles,” such that expression of a large number of genes is measured in each sample (typically >10,000 genes).

**Step 3:** Implement the corresponding diagnostic model for a gene signature identified in Step 1 by following the Methods section in the paper describing the gene signature.

**Step 3A:** If needed, retrain a diagnostic model. We found that some signatures were missing required information in the corresponding paper for us to evaluate those signatures as “locked” models in independent cohorts. For example, when coefficients for a signature based on logistic regression or linear discriminant analysis were missing, we used the discovery cohort to learn the coefficients.

**Step 3B:** If needed, identify a comparable dataset for retraining. This step was required for a subset of signatures because the data on which they were originally trained were not available.

**Step 4:** “Lock” each diagnostic model and apply it to the transcriptome datasets identified in Step 2.

**Step 5:** Aggregate AUROC, specificity at 90% sensitivity, and NPV for each signature across independent datasets, while excluding the corresponding discovery datasets for each signature.

**Step 5A:** Aggregate AUROC, specificity at 90% sensitivity, and NPV using only datasets where diagnosis of ATB is culture-confirmed.

**Step 5B:** Aggregate AUROC, specificity at 90% sensitivity, and NPV using all datasets irrespective of how ATB is diagnosed (culture-confirmed or clinical diagnosis).

**Step 6:** Identify gene signatures that meet the WHO TPP of 70% specificity at 90% sensitivity for a non-sputum-based triage test.

**Step 7:** Aggregate PPV and NPV at 2% prevalence for each signature across independent datasets, while excluding the corresponding discovery datasets for each signature.

**Step 7A:** Aggregate PPV and NPV using only datasets where diagnosis of ATB is culture-confirmed.

**Step 7B:** Aggregate PPV and NPV using all datasets irrespective of how ATB is diagnosed (culture-confirmed or clinical diagnosis).

**Step 8:** Identify gene signatures that meet the FIND TPP of 5.8% PPV at 2% prevalence for a test for predicting progression from LTBI to ATB.

**Step 9:** Identify gene signatures that meet the WHO TPP of >75% specificity and >75% sensitivity for a test for predicting progression from LTBI to ATB.

In response to the comments by the reviewers, we performed bivariate meta-analysis to assess DOR and its heterogeneity for each gene signature, along with FPR.

### Gene signatures for comparison

In June 2018 we performed an extensive search of published gene signatures for distinguishing patients with ATB from healthy controls or patients with LTBI or ODs. We searched the National Center for Biotechnology Information (NCBI) repository of publications (PubMed) for all publications describing a gene signature for the diagnosis of ATB. We included all blood-based gene signatures that were specifically designed to diagnose ATB. We did not exclude any studies because of study criteria or date. Search terms included the following: TB (tuberculosis) gene signature, TB (tuberculosis) transcriptional signature, and TB (tuberculosis) diagnostic. We identified 11 publications describing 16 gene signatures for the diagnosis of ATB (Table 1) [17–27]. We note that we only considered gene signatures that were described for diagnosis of ATB compared to healthy controls or patients with LTBI or ODs. For instance, we did not include the 4-gene RISK4 signature by Suliman et al. [29] or the 16-gene correlates of risk signature by Zak et al. [28] as both signatures are designed to predict progression from LTBI to ATB, not to diagnose ATB as a triage test.

**Table 1:** Gene expression signatures compared within this study.

Citation	PubMed PMID	GEO discovery dataset	Signature name	Indication	Number of genes	Statistical model	Retraining required
Anderson et al. [19]	24785206	GSE39940	Anderson42	ATB vs LTBI	42	Difference of sums	No
			Anderson51	ATB vs ODs	51	Difference of sums	No
Berry et al. [20]	20725040	GSE19491	Berry393	ATB vs (LTBI & HCs)	393	*K*-nearest neighbors	Yes
			Berry86	ATB vs ODs	86	*K*-nearest neighbors	Yes
Bloom et al. [21]	23940611	GSE42834	Bloom144	ATB vs (ODs & HCs)	144	Support vector machine	Yes
Laux da Costa et al. [22]	26025597	GSE42834*	daCosta3	ATB vs ODs	3	Random forest	Yes
Jacobsen et al. [23]	17318616	GSE6112*	Jacobsen3	ATB vs LTBI	3	Linear discriminant analysis	Yes
Kaforou et al. [18]	24167453	GSE37250	Kaforou27	ATB vs LTBI	27	Difference of means	No
			Kaforou44	ATB vs ODs	44	Difference of means	No
			Kaforou52	ATB vs (LTBI & ODs)	52	Difference of means	No
Leong et al. [24]	29559120	GSE101705	Leong24	ATB vs LTBI	24	Rigid logistic regression	Yes
Maertzdorf et al. [25]	26682570	GSE74092	Maertzdorf15	ATB vs (LTBI & HCs)	15	Random forest	Yes
			Maertzdorf4	ATB vs (LTBI & HCs)	4	Random forest	Yes
Sambarey et al. [26]	28065665	GSE37250*	Sambarey10	ATB vs (LTBI & HCs & ODs)	10	Linear discriminant analysis	Yes
Sweeney et al. [17]	26907218	GSE19491, GSE37250, GSE42834	Sweeney3	ATB vs (LTBI & ODs & HCs)	3	Difference of geometric means	No
Verhagen et al. [27]	23375113	GSE41055	Verhagen10	ATB vs (LTBI & HCs)	10	Random forest	Yes

Gene signatures are named by combining the last name of the first author followed by the number of genes in the signature.

*The diagnostic model for the signature was created using this dataset as the original training dataset was not available.

ATB, active tuberculosis; GEO, Gene Expression Omnibus; HC, healthy control; LTBI, latent tuberculosis infection; OD, other disease.

### Recreating corresponding classification models for each gene signature

For each of the 16 published signatures, we constructed a classification model as described in the original paper to the best of our ability. We created and trained each classification model to be as accurate a replica of the model in the original publication as possible, using the same datasets used in the original publication except where we were unable to access the original training data (Table 1). In those instances, we trained the model on a different, suitable dataset as indicated in Table 1. We confirmed that the classification models were successfully reconstructed by comparing the performance of each model to the performance of the model as described in the original publication (S1 Table). S1 Text provides a detailed description of each model.

### Transcriptome datasets used for comparing signatures

We searched NCBI GEO and European Bioinformatics Institute (EBI) ArrayExpress in June 2018 using the following search terms: TB (tuberculosis) gene expression, TB (tuberculosis) microarray, TB (tuberculosis) blood microarray, TB (tuberculosis) RNAseq, TB (tuberculosis) blood RNAseq, TB (tuberculosis) peripheral blood mononuclear cells gene expression, TB (tuberculosis) peripheral blood mononuclear cells microarray, and TB (tuberculosis) peripheral blood mononuclear cells RNAseq. We included all datasets that measured transcriptomes from the blood of patients with ATB and at least 1 other group of individuals. We did not exclude datasets based on collection date or sample number. We excluded datasets profiled using quantitative PCR because they did not have enough coverage to capture all genes across the 16 signatures included in our analysis. We identified 24 datasets containing 3,083 transcriptome profiles from whole blood or PBMCs of patients with ATB and healthy controls or patients with LTBI or ODs from 14 countries through an extensive search of 2 public data repositories (NCBI GEO and EBI ArrayExpress) (Table 2). These datasets also include the 8 datasets that were used to derive the 16 gene signatures. We used these 24 datasets to compare each of the 16 gene signatures for their ability to distinguish ATB from all other groups (healthy controls, LTBI, and ODs). In most instances the method of diagnosis of patients with ATB could be confirmed (as indicated under “diagnosis method” in Table 2); in some cases the method of diagnosis could not be identified from the information available publicly. The datasets included in this analysis were collected in 14 different countries and measures on 18 different platforms. In total these datasets include 3,083 individuals, of whom 944 were patients with ATB at the time of sample collection.

**Table 2:** Transcriptome datasets used for comparison of 16 gene signatures for diagnosis of ATB.

GEO accession	GEO platform	Country	Tissue	Age (years)	HIV status	Diagnosis method	HCs	LTBI	ATB	ODs	Total	Notes
GSE19491	GPL6947	UK, South Africa	Whole blood	>17	Negative	Sputum culture	117	69	61	193	440	ODs included staph, strep, Still disease, systemic lupus erythematosus, and pediatric systemic lupus erythematosus
GSE28623	GPL4133	The Gambia	Whole blood	16–53	Negative	Sputum microscopy, chest X-ray	37	25	46		108	
GSE29536	GPL6102	UK	Whole blood	11–88	Negative		6		9		15	Only the TB dataset within this series was used
GSE34608	GPL6480	Germany	Whole blood	17–73	Negative		18		8	18	108	OD samples were sarcoidosis samples; controls may have included some IGRA-positive individuals
GSE37250	GPL10558	Malawi	Whole blood	>17	Some positive	Sputum culture		167	195	175	537	
GSE39939	GPL10558	Kenya	Whole blood	<15	Some positive	Sputum culture		14	79	64	157	
GSE39940	GPL10558	Malawi	Whole blood	<15	Some positive	Sputum culture		54	111	169	334	
GSE41055	GPL5175	Venezuela	Whole blood	<15	Negative		9	9	9		27	
GSE42834	GPL10558	Germany	Whole blood	>17	Negative	Sputum culture	118		40	123	281	ODs included sarcoidosis, pneumonia, and lung cancer
GSE50834	GPL10558	South Africa	PBMCs	30–40	Positive			23	21		44	
GSE56153	GPL6883	Indonesia	Whole blood	>15	Negative	Sputum microscopy, chest X-ray, clinical presentation	18		18		36	HIV status not measured in controls, but HIV has very low prevalence in Indonesia
GSE54992	GPL570	China	PBMCs	18–68	Negative	Sputum microscopy, chest X-ray, clinical presentation, sputum culture	6	6	9		21	Dataset also included 18 treated samples not used in this analysis; samples confirmed with sputum culture were not identified
GSE62147	GPL6480	Germany	Whole blood	15–79	Negative	Sputum culture			14	12	26	OD was *Mycobacterium africanum*
GSE62525	GPL16951	Taiwan	PBMCs		Negative		14	14	14		42	
GSE69581	GPL10558	South Africa	Whole blood	>17	Positive	Microbiologically confirmed (method not specified)		25	15		40	Dataset also included samples with subclinical TB
GSE73408	GPL11532	US	Whole blood	>17	Negative	Sputum culture	39	35	35		109	
GSE79362	GPL11154	South Africa	Whole blood	12–18	Negative	Sputum smear, sputum culture		101	19		120	Longitudinal samples were collected
GSE81746	GPL17077	India	Whole blood	25–65	Unknown		2		4		6	
GSE83456	GPL10558	UK	Whole blood		Negative	Granuloma biopsy, clinical presentation with response to therapy, radiology with response to treatment, sputum culture	61		45	49	155	ODs included sarcoidosis; there were also extrapulmonary TB samples available from this dataset.
GSE83892	GPL10559	UK	Whole blood	>17	Positive	Cerebral spinal fluid smear, cerebral spinal fluid culture, conventional and real-time PCR of cerebral spinal fluid		17	99		116	ATB patients in this cohort included patients with complications from immune reconstitution inflammatory syndrome and TB meningitis
GSE84076	GPL16791	Brazil	Whole blood	>18	Negative	Sputum microscopy, clinical presentation, sputum culture	6	6	9		21	This dataset also included some samples taken from individuals after treatment
GSE101705	GPL18573	India	Whole blood	>6	Negative	Sputum culture		16	28		44	
GSE107731	GPL15207	Mongolia	Whole blood		Unknown		3		3		6	
GSE107994	GPL20301	UK	Whole blood	16–84	Negative	Sputum culture, sputum PCR	119	118	53		290	Dataset included samples from latent TB progressors
**24 Datasets**		**14 countries**					**573**	**699**	**944**	**803**	**3,083**	

ATB, active tuberculosis; GEO, Gene Expression Omnibus; HC, healthy control; IGRA, Interferon Gamma Release Assay; LTBI, latent tuberculosis infection; OD, other disease; PBMC, peripheral blood mononuclear cell; TB, tuberculosis.

### Evaluation of model performance

We assessed the performance of each signature in each dataset using AUROC statistics that were calculated for each model across all datasets not used in the discovery or training of the model, as well as across only culture-confirmed cases not used in the discovery or training of the model. In an effort to avoid bias against any model, the AUROC was calculated at the optimal cut-point for each dataset/model combination. We used the R package OptimalCutpoints to determine the AUROC and Youden index for each signature in each dataset. The optimal cut-point was identified using the Youden method [33]. A weighted mean AUROC was then calculated for each model. No universal cutoffs were used, but rather the most favorable cutoff for each model under each condition.

### Weighted mean statistics

The weighted mean AUROC was calculated as shown below in Eq 1, where *d* is the number of datasets analyzed, AUROC_*i*_ is the AUROC for a given dataset *i*, and *n*_*i*_ is the number of samples within dataset *i* [34]:

$$\text{weighted mean AUROC}=\frac{\sum_{i=1}^{d}{\text{AUROC}}_{i}*n_{i}}{\sum_{i=1}^{d}n_{i}}$$ (1)

The 95% confidence interval for the weighted mean AUROC was calculated by adding and subtracting 1.96 times the standard deviation (SD) of the mean from the weighted mean AUROC according to Eqs 2 and 3 below, where ω refers to the weighted sum of observations squared, σ refers to the sum of observations squared, and δ refers to the degrees of freedom:

$$\text{SD}=\;\sqrt[{2}]{\frac{\left(\omega -\sigma \right)}{\left(\delta \right)}}$$ (2)

95%CI=weighted mean AUROC±(1.96*SD) (3)

## Results

Our systematic search of the literature for published transcriptional signatures diagnosing ATB against other clinical conditions identified 16 transcriptional signatures (Table 1) that distinguished patients with ATB from 1 or more of the following: healthy controls, patients with LTBI, or patients with ODs. Next, we searched 2 public data repositories (NCBI GEO and EBI ArrayExpress) for gene expression datasets that profiled whole blood or PBMC samples comparing patients with ATB to healthy controls or patients with LTBI or ODs. We identified 24 independent datasets consisting of 3,083 transcriptome profiles from 14 countries (Table 2). Note that 8 of these 24 datasets were used to derive 1 or more of the 16 gene signatures. Therefore, in order to ensure that the discovery cohort(s) of each signature did not bias its overall performance, we removed the corresponding discovery cohort(s) for each signature when computing the overall performance of each signature across all datasets. For example, for the Verhagen10 signature, we removed GSE41055 when estimating the overall AUROC and PPV, whereas for Sweeney3 we removed GSE19491, GSE37250, and GSE42834.

**Fig 1:**
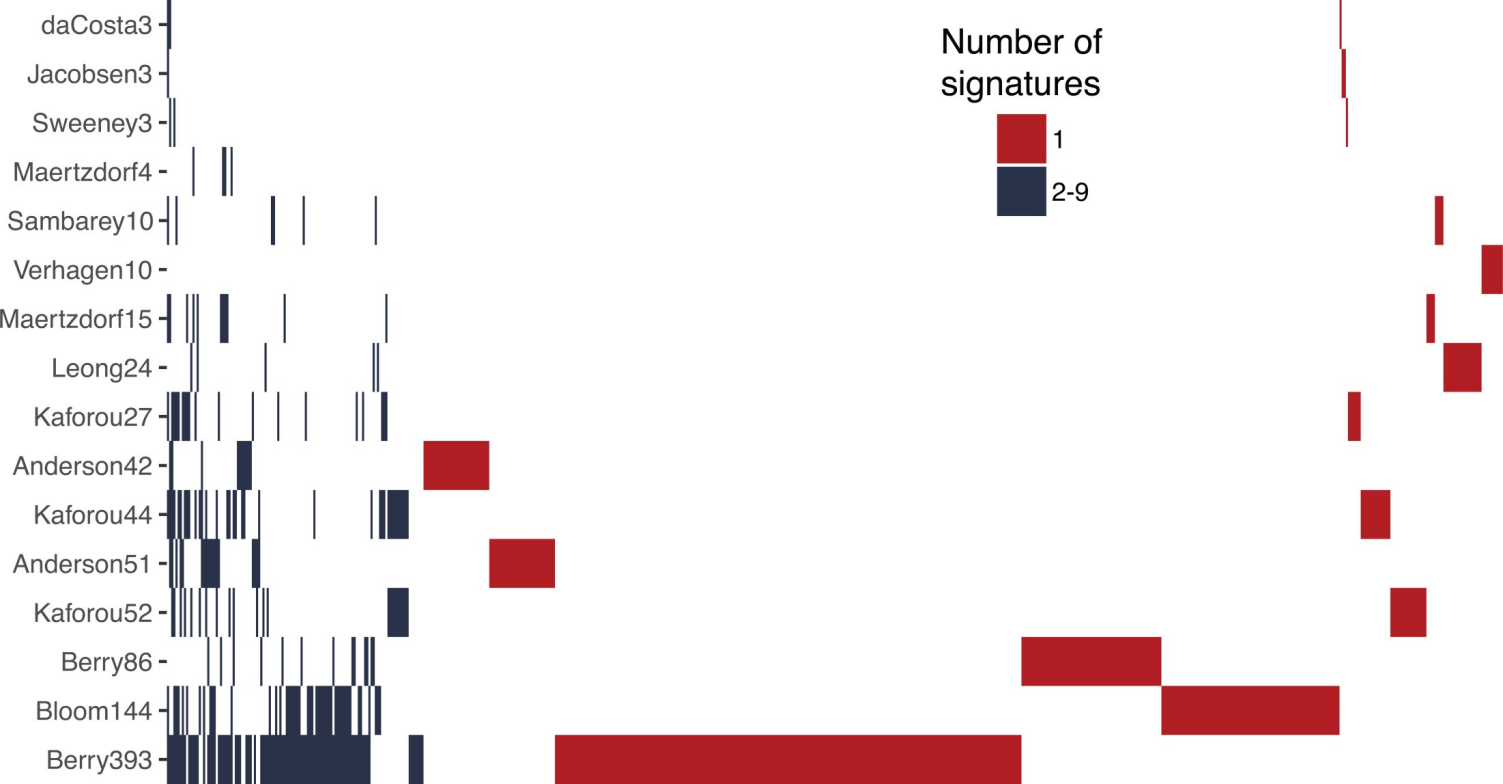
Distribution of genes across the signatures included in this study. Each row represents a gene signature for active tuberculosis diagnosis. Each column represents 1 gene. The number at the end of a signature name represents the number of genes in the given signature. Genes present in only 1 signature are red; those in 2 or more signatures are blue.

Overall, 630 genes were described across the 16 signatures, with the number of genes in a signature ranging from 3 [17,22,23] to 393 [20] (Fig 1). A majority of the genes (81%) were in only 1 signature. Every gene signature partially overlapped with at least 1 other signature. One gene signature (Maertzdorf4) did not include a unique gene as it was derived from another gene signature (Maertzdorf15).

### Comparison of accuracy across only datasets with culture-confirmed diagnosis of ATB

For each of the 16 gene signatures, we built a classification model by following the methods described in the corresponding paper to the best of our ability. We found that the classification model we created for each signature closely reproduced the AUROC reported in the corresponding paper, though not exactly (S1 Table). We applied these “locked” models “as is” to other datasets to assess the generalizability of each model for accurately distinguishing patients with culture-confirmed ATB across independent datasets.

Across the 9 independent datasets with culture-confirmed ATB, 11 gene signatures had weighted mean AUROC > 0.8, which suggests that many of the host-response-based signatures tended to have generalizability (Table 3). However, 2 gene signatures (Verhagen10 and Anderson51) had weighted mean AUROC ≤ 0.6. All but 2 gene signatures (Berry86 and Berry393) had high NPV (>98% at 2% prevalence) in culture-confirmed datasets. Signatures with low AUROC or NPV (Verhagen10, Leong24, Anderson51, Berry86, and Berry393) suggest that several signatures did not generalize to independent cohorts with culture-confirmed diagnosis of ATB. Arguably, the lack of generalizability in these signatures may be expected for several reasons. First, some signatures were derived using samples from children (Verhagen10 and Anderson51). Second, a signature may not have been derived to be generalizable. For instance, Anderson et al. [19] described 2 gene signatures (Anderson42 and Anderson51) from the same dataset of children under 5 years of age for distinguishing patients with ATB from patients with LTBI (Anderson42) or with ODs (Anderson51), which may not generalize to adults. In contrast, the Sweeney3 signature, which was derived using 3 independent cohorts of adults, generalized to children (age ≤ 5 years) and adolescents (age 12–18 years). Across all culture-confirmed datasets, the Sweeney3 and Sambarey10 signatures had the highest accuracy in distinguishing patients with ATB from healthy controls and patients with LTBI or ODs (specificity 74% at 90% sensitivity). Both signatures were the only signatures to meet the minimal WHO TPP for a triage test in cohorts of patients with culture-confirmed diagnosis of ATB.

**Table 3:** Weighted mean AUROC, specificity at 90% sensitivity, and NPV at 2% prevalence for ATB versus all other conditions across all datasets and across only culture-confirmed datasets for each of the 16 gene signatures.

Signature	Culture-confirmed datasets			All datasets		
	AUROC (95% CI)	Specificity (95% CI)	NPV	AUROC (95% CI)	Specificity (95% CI)	NPV
Sweeney3	0.89 (0.82–0.96)	0.74 (0.40–0.89)	0.99	0.85 (0.72–0.99)	0.66 (0.23–0.93)	0.98
Jacobsen3	0.86 (0.72–1.00)	0.68 (0.37–0.93)	0.99	0.83 (0.69–0.98)	0.59 (0.21–0.92)	0.99
daCosta3	0.83 (0.60–1.00)	0.65 (0.31–0.88)	0.99	0.76 (0.45–1.00)	0.50 (0.00–0.95)	0.94
Maertzdorf4	0.83 (0.74–0.91)	0.58 (0.28–0.82)	0.99	0.79 (0.64–0.95)	0.54 (0.24–0.79)	0.99
Sambarey10	0.90 (0.83–0.97)	0.74 (0.36–0.94)	0.99	0.82 (0.57–1.00)	0.59 (0.18–0.94)	0.99
Verhagen10	0.53 (0.46–0.60)	0.13 (0.11–0.19)	0.98	0.54 (0.41–0.68)	0.14 (0.00–0.32)	0.92
Maertzdorf15	0.82 (0.71–0.92)	0.58 (0.30–0.82)	0.99	0.79 (0.66–0.92)	0.54 (0.23–0.83)	0.99
Leong24	0.74 (0.53–0.95)	0.41 (0.12–0.63)	0.99	0.75 (0.54–0.95)	0.43 (0.04–0.77)	0.99
Kaforou27	0.86 (0.71–0.92)	0.66 (0.40–0.92)	0.99	0.83 (0.64–1.00)	0.62 (0.28–0.94)	0.99
Anderson42	0.84 (0.75–0.93)	0.61 (0.39–0.82)	0.99	0.82 (0.66–0.97)	0.58 (0.27–0.87)	1.00
Kaforou44	0.82 (0.67–0.97)	0.61 (0.27–0.80)	0.99	0.78 (0.56–1.00)	0.54 (0.12–0.85)	0.99
Anderson51	0.60 (0.42–0.79)	0.22 (0.00–0.44)	0.99	0.58 (0.33–0.82)	0.21 (0.00–0.52)	0.96
Kaforou52	0.87 (0.77–0.97)	0.67 (0.45–0.87)	0.99	0.84 (0.70–0.99)	0.62 (0.29–0.92)	0.99
Berry86	0.67 (0.44–0.90)	0.21 (0.00–0.76)	0.47	0.69 (0.36–1.00)	0.29 (0.00–0.65)	0.47
Bloom144	0.81 (0.61–1.00)	0.50 (0.10–0.64)	0.99	0.74 (0.52–0.96)	0.33 (0.00–0.65)	0.98
Berry393	0.72 (0.43–1.00)	0.40 (0.00–1.00)	0.74	0.71 (0.43–0.99)	0.34 (0.00–0.98)	0.66

ATB, active tuberculosis; AUROC, area under the receiver operating characteristic curve; NPV, negative predictive value.

### Comparison of accuracy across all datasets for diagnosis of ATB

Next, we compared accuracy of the 16 signatures across all datasets irrespective of how ATB was diagnosed, which included sputum microscopy and clinical presentation (Table 3). Weighted mean AUROC for all but 3 signatures was lower than when only considering culture-confirmed datasets, although these decreases were not meaningful (≤4% decrease). Importantly, 3 signatures (Sambarey10, daCosta3, and Bloom144) had a substantial reduction in AUROC (7%, 8%, and 7%, respectively), which results in their specificity at 90% sensitivity decreasing by greater than 15%. None of the gene signatures met the minimal WHO TPP for the triage test when including all datasets. The AUROC for the Sweeney3 gene signature decreased by 4%, resulting in an overall specificity of 66% at 90% sensitivity, which was the highest among all signatures when datasets were included irrespective of how ATB was diagnosed. Another signature (Kaforou52) also had specificity 62% at 90% sensitivity, but included 52 genes, a substantially higher number of genes than the Sweeney3 signature. Notably, we found no correlation between the number of genes in a signature and the weighted mean AUROC (*R* = −0.04, *p* = 0.86). Finally, 5 gene signatures (daCosta3, Verhagen10, Anderson51, Berry86, and Berry393) had NPV (<98%) too low to be clinically useful as a triage test.

### Comparison of accuracy for diagnosis of ATB using bivariate meta-analysis

The comparison of the 16 signatures can also be performed as a bivariate meta-analysis by combining sensitivity and specificity from diagnostic tests across different datasets. We used the R package mada to compare the 16 signatures by computing their DOR, heterogeneity in DOR, and overall FPR. A clinically useful generalizable triage test should have low FPR and high DOR with no heterogeneity across characteristics of patient populations such as genetic background of host, *Mtb* strain, age, HIV co-infection, and bacillus Calmette–Guérin vaccination.

Four signatures (Sweeney3, Kaforou52, Kaforou44, and Anderson51) had no heterogeneity irrespective of what datasets were used for analysis (only culture-confirmed or all datasets); the remaining 12 signatures showed heterogeneity (Table 4). Among the 4 signatures with no heterogeneity, the Sweeney3 signature had the highest DOR with the lowest FPR (Table 4) irrespective of what datasets were used, and was the only signature using fewer than 10 genes. Interestingly, when only culture-confirmed datasets were used, the daCosta3 signature had the highest DOR (32.44), with no heterogeneity and a 26% FPR. However, when datasets with clinical diagnoses of ATB were included, the daCosta3 signature had increased heterogeneity (13.63%) and a very high FPR (45%). On the other hand, the Maertzdorf15 signature had substantial heterogeneity (19.07%) when using only culture-confirmed datasets, which decreased to no heterogeneity when datasets with clinical diagnoses were included, without substantial changes in DOR or FPR. These changes in heterogeneity and FPR depending on which datasets are used in the analysis further suggest that certain signatures may not be generalizable to broad patient populations.

**Table 4:** Comparison of 16 gene signatures for diagnosis of ATB using bivariate meta-analysis.

Signature	Culture-confirmed datasets			All datasets		
	DOR (95% CI)	Heterogeneity	FPR (95% CI)	DOR (95% CI)	Heterogeneity	FPR (95% CI)
Sweeney3	30.50 (14.95–62.24)	0	0.18 (0.13–0.26)	16.66 (11.56–24.00)	0	0.20 (0.16–0.24)
Kaforou52	21.05 (12.20–36.34)	0	0.23 (0.16–0.33)	14.05 (10.10–19.54)	0	0.23 (0.18–0.28)
Kaforou44	12.22 (6.04–24.71)	0	0.22 (0.16–0.30)	9.05 (6.42–12.74)	0	0.22 (0.17–0.29)
Anderson51	4.96 (2.86–8.59)	0	0.26 (0.09–0.53)	3.91 (2.90–5.26)	0	0.32 (0.20–0.46)
Jacobsen3	19.89 (10.72–36.89)	4.03	0.22 (0.16–0.30)	13.04 (9.54–17.82)	0	0.21 (0.17–0.26)
Kaforou27	17.21 (11.08–26.74)	1.13	0.25 (0.17–0.34)	13.85 (10.32–18.59)	0	0.23 (0.18–0.29)
Maertzdorf15	14.38 (8.04–25.70)	19.07	0.27 (0.21–0.34)	11.65 (8.37–16.22)	0	0.26 (0.20–0.31)
Maertzdorf4	13.82 (8.75–21.83)	0.73	0.24 (0.18–0.31)	9.69 (7.39–12.71)	3.06	0.28 (0.23–0.33)
Anderson42	11.26 (7.50–16.92)	4.8	0.28 (0.24–0.33)	10.65 (7.87–14.42)	8.39	0.26 (0.21–0.31)
Sambarey10	19.13 (10.38–35.25)	16.87	0.19 (0.13–0.28)	12.18 (8.54–17.37)	11.61	0.20 (0.17–0.24)
daCosta3	32.44 (14.90–70.63)	0	0.26 (0.13–0.44)	13.89 (8.14–23.71)	13.63	0.45 (0.28–0.64)
Verhagen10	1.85 (1.30–2.63)	21.43	0.47 (0.28–0.67)	2.90 (2.03–4.15)	21.02	0.47 (0.31–0.65)
Bloom144	9.94 (5.49–17.99)	50.55	0.21 (0.13–0.32)	6.69 (4.71–9.49)	23.29	0.24 (0.16–0.34)
Leong24	8.20 (4.75–14.16)	46.33	0.27 (0.18–0.39)	8.48 (5.96–12.06)	23.93	0.26 (0.19–0.34)
Berry393	17.72 (7.41–42.35)	33.39	0.16 (0.09–0.27)	9.26 (5.90–14.53)	25.48	0.45 (0.25–0.66)
Berry86	12.62 (4.98–31.99)	42.89	0.19 (0.04–0.57)	6.72 (3.81–11.85)	27.48	0.66 (0.35–0.87)

ATB, active tuberculosis; DOR, diagnostic odds ratio; FPR, false positive rate.

### Signature performance predicting progression 6 months prior to ATB diagnosis

Predicting progression from LTBI to ATB prior to sputum conversion is an important step in reducing overall incidence of ATB. Previously, a 16-gene (CoR [28]) and a 4-gene (RISK4 [29]) signature have been described to identify individuals with a high likelihood of progression. CoR was derived from and validated in the Adolescent Cohort Study (ACS; GSE79362) with validation AUROC = 69% with 66% sensitivity and 81% specificity. However, this signature was shown to have poor generalizability in the GC6-74 cohort from other African countries in a follow-up study. Therefore, we excluded CoR from our comparison. The second signature, RISK4, was derived from the GC6-74 cohort and was shown to have AUROC = 69% in the ACS [29]. Using the ACS, we compared the 16 gene signatures for their ability to predict progression from LTBI to ATB 6 months prior to sputum conversion. Because CoR was derived from the ACS cohort and shown to be not generalizable to cohorts from other African countries, we excluded the CoR signature from the comparison. We also excluded the RISK4 signature from further comparison as its performance characteristics are previously described in the ACS cohort [29].

Seven out of 16 signatures had AUROC > 0.8 and PPV > 5.8% at 2% prevalence in the ACS cohort (Table 5). Higher numbers of genes in a signature again did not correspond to a substantial increase in the AUROC. Only 2 of these signatures (Sweeney3 and Jacobsen3) had fewer than 10 genes. Interestingly, although the daCosta3 signature had the highest PPV (14.60%) for predicting progression from LTBI to ATB, it had AUROC = 0.56. Sweeney3 had the lowest number of genes with the highest AUROC (0.86, 95% CI 0.78–0.93) and PPV (13.6%), which exceeded the FIND TPP for the progression test. Overall, these results demonstrated that, in patients with LTBI, host response is able to identify those at high risk of progression to ATB prior to sputum conversion.

**Table 5:** AUROC, PPV, and NPV for progression from LTBI to ATB in the ACS cohort up to 180 days prior to diagnosis.

Signature	AUROC (95% CI)	PPV at 2% prevalence	NPV at 2% prevalence
daCosta3	0.56 (0.50–0.62)	14.60	98.2
Sweeney3	0.86 (0.78–0.94)	13.60	99.4
Kaforou27	0.86 (0.78–0.94)	13.60	99.4
Kaforou52	0.87 (0.80–0.95)	13.20	100
Leong24	0.73 (0.62–0.83)	11.00	99.3
Jacobsen3	0.85 (0.76–0.93)	10.80	NC
Anderson42	0.85 (0.77–0.92)	8.80	99.5
Bloom144	0.68 (0.56–0.79)	8.30	98.9
Sambarey10	0.80 (0.72–0.89)	6.40	99.4
Kaforou44	0.83 (0.76–0.90)	6.20	99.4
Maertzdorf15	0.63 (0.56–0.69)	2.80	99.5
Anderson51	0.46 (0.34–0.58)	2.70	98.2
Maertzdorf4	0.51 (0.50–0.52)	2.00	99.5
Verhagen10	0.47 (0.45–0.49)	2.00	NC
Berry86	0.50 (0.50–0.50)	2.00	98.9
Berry393	0.50 (0.50–0.50)	2.00	NC

ACS, Adolescent Cohort Study; ATB, active tuberculosis; AUROC, area under the receiver operating characteristic curve; LTBI, latent tuberculosis infection; NC, not calculated; NPV, negative predictive value; PPV, positive predictive value.

## Discussion

In this study, we compared 16 gene signatures for distinguishing patients with ATB from healthy controls or patients with LTBI or ODs using 24 independent datasets of >3,000 whole blood or PBMC transcriptome profiles from 14 countries. Collectively, these datasets represented real-world heterogeneity observed in patients with TB. For instance, the samples collected across 14 countries represented diversity in both host and pathogen genetics. Similarly, some datasets profiled samples from children whereas others profiled samples from adults, which represented heterogeneity in host response due to age. These data also represented heterogeneity in clinical practice as patients were diagnosed using different criteria (e.g., sputum culture versus sputum microscopy).

Across these biologically and technologically heterogeneous data, our comparison found that several gene signatures distinguished patients with ATB with moderate to high accuracy, although almost all signatures included a large number of genes, which severely restricts their ability for cost-effective translation to clinical practice at the POC. Importantly, our analysis found that a higher number of genes in a signature did not translate into higher accuracy across biologically heterogeneous data. Only 2 gene signatures (Sweeney3 and Sambarey10) satisfied the WHO TPP for a non-sputum-based triage test to identify which patients need further testing for confirming ATB, when comparing the signatures using only datasets from patients with culture-confirmed diagnosis of ATB.

When we included additional datasets that diagnosed ATB using other means (e.g., sputum microscopy or clinical diagnosis), the accuracy of 13 signatures decreased such that no signature satisfied the WHO TPP for a non-sputum-based triage test. For the 2 signatures that satisfied the WHO TPP for a non-sputum-based triage test when using only culture-confirmed datasets, the reduction in the AUROC and specificity of Sweeney3 was minimal (AUROC = 0.85, specificity = 66%, and sensitivity = 90%), whereas Sambarey10 had a substantial reduction of 15% in specificity. It is possible that inclusion of patients with ATB that was not diagnosed using positive culture caused the reduction in accuracy and may underestimate the accuracy of these signatures.

The inclusion of children in our analyses could have decreased the overall performance regarding misclassification of cases because of the challenges in diagnosis of ATB in children. GSE39939 and GSE39940, both of which were part of the same study [19], contained 157 and 334 samples (491 samples total), respectively, suggesting that the number of children in these datasets was sufficient. Out of the 491 samples, 190 samples were from children with ATB, of which 44 patients with ATB were sputum-negative; the remaining 146 children with ATB were sputum-positive. When using children with sputum-positive ATB, we did not see a substantial decrease in overall performance compared to adults. However, when children with clinically diagnosed or culture-negative ATB were included in our analysis, every gene signature had lower accuracy, similar to when including adults with clinically diagnosed or culture-negative ATB. These results suggest that gene signatures for diagnosis of ATB based on host response are not substantially affected by age, but by the possible inaccuracy of culture-negative clinical diagnosis of ATB.

We found that 7 signatures (Sweeney3, Kaforou27, Kaforou52, Jacobsen3, Anderson42, Sambarey10, and Kaforou44) identified adolescents with LTBI who progressed to ATB up to 6 months prior to sputum conversion. Among these signatures, only Sweeney3 has been prospectively validated in an active screening cohort to further demonstrate that host response to *Mtb* is detectable in blood samples earlier [13]. These results further suggest that host-response-based gene signatures could have substantial impact on the diagnosis of incipient TB, which is defined as an asymptomatic phase with early disease [35]. Incipient TB may last for up to 1 year approximately, during which a patient may be intermittently infectious by shedding bacilli in the sputum. Although the exact definitions of LTBI and incipient TB may be different, patients with LTBI progressing towards ATB in principle are similar to patients with incipient TB. Our results suggest that host-response-based gene signatures should be further explored for diagnosis of incipient TB in larger cohorts.

The ability of gene signatures for diagnosis of ATB to predict progression from LTBI to ATB prior to sputum conversion and to diagnose ATB in active screening is additional indirect evidence that suggests our estimates of accuracy for each gene signature may be underestimates. Many of the samples labeled as LTBI but classified as ATB may be progressors with subclinical ATB. Collectively, these results highlight the need for assessing the host-response-based TB diagnostics in larger prospective cohorts.

An important contributing factor to the lower generalizability of several signatures is likely the choice of underlying classification model. For instance, signatures using *K*-nearest-neighbors clustering as a model had the overall worst performance because of the lack of co-normalization of data across datasets and platforms. Models based on *K*-nearest-neighbors clustering benefit from co-normalized data; however, it is impractical, and may be very difficult, if not nearly impossible, to co-normalize data from different clinics using different technologies.

Overall, when considering the feasibility of translating a gene signature as a POC test (e.g., number of genes, required specificity at 90% sensitivity, and robustness across datasets from different geographic regions and clinical contexts), the Sweeney3 signature consistently ranked among the best signatures. The signature has also been prospectively validated in at least 2 independent cohorts using reverse transcription PCR [11,30]. Collectively, the Sweeney3 signature has been now shown to (1) predict progression to ATB 6 months prior to sputum conversion, (2) distinguish ATB in active screen, (3) track treatment response, and (4) stratify patients with ATB at the time of diagnosis with high likelihood of subclinical ATB after treatment. This robustness of Sweeney3 may be due to the fact that it was derived using 3 independent cohorts that represented broad biological and technical heterogeneity. This is in line with the observation by Suliman et al. [29] that a gene signature derived using a homogeneous cohort from 1 country was not broadly applicable to patients from other countries with similar genetic background. It is important to note that Sweeney3 is the only prospectively validated signature among the 16 signatures compared here. Importantly, the ability of the Sweeney3 gene signature to identify patients with ATB in different clinical contexts and achieve the WHO TPP using only 3 genes provides strong evidence for the potential of host-response-based diagnostics to impact clinical practice.

Our analysis has a few limitations. First, we did not have access to the exact published models, or any hyper-parameters used to build the models, for some of the gene signatures compared here. Therefore, we re-implemented these models to the best of our ability by following the details in the corresponding papers. Hence, in the process of trying to build models that reproduced as closely as possible the results reported in the corresponding paper, the choices we made and hyper-parameters we inferred may have been different from the those in the original models. This could have resulted in overfitting, which in turn may have resulted in reduced generalizability of models in independent cohorts and underestimation of their accuracy. We recommend that when diagnostic signatures are published, the corresponding models should be made available, along with a list of hyper-parameters and coefficients to enable reproducibility and comparison between models. Second, for some studies we were not able to use the original training data as they were not available. We chose another dataset that was similar to the discovery cohort described in the corresponding paper. This choice again may have resulted in underestimation of accuracy. Therefore, if a model was extremely sensitive to training data, overfitting may have happened. This limitation points to the need for sharing underlying data used for building a classification model. Third, none of the datasets used in our analysis included patients with nontuberculous mycobacteria infections. Therefore, it is not possible to evaluate whether the signatures compared here can differentiate patients with nontuberculous mycobacteria infections or ATB. Fourth, LTBI was defined using either a tuberculin skin test (TST) or IGRA, which could have different implications for progression to ATB. It is possible that patients identified as having LTBI using different diagnostic criteria could have different transcriptome profiles. Our results showed that a few gene signatures demonstrated consistently high accuracy across datasets irrespective of how LTBI was defined, suggesting that host response to ATB is sufficiently different and robust to overcome the heterogeneity in clinical practice of how LTBI is defined. Importantly, our work described here points to future studies of how existing data could be used to identify differences in the transcriptomes of patients with LTBI diagnosed with TST or IGRA.

Despite the limitations in re-implementation of current ATB diagnostic models, our comparison of 16 blood-based gene signatures strongly suggests the potential of using host-response-based gene signatures for diagnosis of ATB. The fact that a subset of signatures perform with clinically useful accuracy across multiple datasets with no heterogeneity further suggests that they should be explored in larger prospective cohorts for estimating their impact on clinical practice, instead of creating more gene signatures. Further studies should compare these signatures to investigate whether they are correlated with each other (identify the same patients or miss the same patients). If different signatures correctly diagnose different patient populations, it may be advisable to integrate these signatures in a single diagnostic model. However, before validating these signatures in prospective cohorts, they must be published and “locked” for other researchers to investigate such as we have done here. It is highly unlikely that any of these signatures will be measured using RNA sequencing or microarrays in resource-poor areas where TB is prevalent. Therefore, the prospective studies for these signatures should be performed using technologies that are cost-effective when used on a large scale. When host-response-based ATB diagnostics are validated in prospective studies, they should be designed to facilitate the identification of a threshold that can be used in clinical practice. Future prospective trials will also have to understand whether 1 threshold is sufficient across different clinical contexts (e.g., progression from LTBI to ATB, active screening for ATB, and treatment response) or multiple, better-tuned thresholds for each clinical context are needed.

### Conclusion

With the increasing number of blood-based signatures for diagnosis of ATB being proposed, it is important to investigate whether measuring host response is appropriate and, if it is, whether any of the existing signatures are able to meet the WHO TPP and should be investigated for translation to clinical practice. We found that when using datasets with only culture-confirmed diagnosis of ATB, only 2 signatures met the minimal WHO TPP for a non-sputum-based triage test. No signature met the minimal WHO TPP when datasets with clinical diagnosis of ATB were included, which may be due to the lower accuracy of clinical diagnoses. Bivariate meta-analysis of these signatures further showed that only 4 out of the 16 gene signatures had no heterogeneity irrespective of which datasets were included in the analysis. Further, we found that 7 signatures met the TPP for a test for predicting progression from LTBI to ATB. Overall, across all comparisons, only the Sweeney3 signature had fewer than 10 genes, met the WHO and FIND TPPs for a non-sputum-based triage test for diagnosis of ATB and predicting progression from LTBI to ATB, and performed robustly with high DOR without heterogeneity and the lowest FPR. We found that higher numbers of genes in a signature did not increase the accuracy of the signature. Our results provide strong evidence for the potential of host-response-based diagnostics in achieving the WHO goal of ending TB by 2035, and should be pursued for clinical implementation.

## Supporting information

S1 PRISMA checklist (DOCX)

S1 Table Comparison of re-implemented classification models for 16 gene signatures with the models in the corresponding original reports. (XLSX)

S1 Text Description of the re-implemented classification models for each signature. (DOCX)

## Abbreviations

ACS: Adolescent Cohort Study
ATB: active tuberculosis
AUROC: area under the receiver operating characteristic curve
DOR: diagnostic odds ratio
EBI: European Bioinformatics Institute
FIND: Foundation for Innovative New Diagnostics
FPR: false positive rate
GEO: Gene Expression Omnibus
IGRA: Interferon Gamma Release Assay
LTBI: latent tuberculosis infection
Mtb: Mycobacterium tuberculosis
NCBI: National Center for Biotechnology Information
NPV: negative predictive value
OD: other disease
PBMC: peripheral blood mononuclear cell
POC: point of care
PPV: positive predictive value
TB: tuberculosis
TPP: target product profile
TST: tuberculin skin test
WHO: World Health Organization
