# Supplementary material for: Host-response-based gene signatures for tuberculosis diagnosis: A systematic comparison of 16 signatures
Source: PLoS Med. 2019 Apr 23;16(4):e1002786. doi: 10.1371/journal.pmed.1002786 (PMC6478271; doi:10.1371/journal.pmed.1002786)
Supplement: S1 PRISMA checklist — (DOCX) [file pmed.1002786.s001.docx]

| **Section/topic** | **#** | **Checklist item** | **Reported on page #** |
| --- | --- | --- | --- |
| **TITLE** | | |  |
| Title | 1 | Identify the report as a systematic review, meta-analysis, or both. |  |
|  |  | A systematic comparison of host response-based gene signatures for diagnosis of tuberculosis | 1 |
| **ABSTRACT** | | |  |
| Structured summary | 2 | Provide a structured summary including, as applicable: background; objectives; data sources; study eligibility criteria, participants, and interventions; study appraisal and synthesis methods; results; limitations; conclusions and implications of key findings; systematic review registration number. |  |
|  |  | Background: The World Health Organization (WHO) and Foundation for Innovative New Diagnostics (FIND) have published target product profiles (TPPs) calling for non-sputum based diagnostic tests for the diagnosis of active tuberculosis disease (ATB) and predicting the progression from latent tuberculosis infection (LTBI) to ATB. A large number of host-derived blood-based gene-expression biomarkers for diagnosis of patients with ATB have been proposed to date, but none have been implemented in clinical settings. The focus of this study is to directly compare published gene signatures for diagnosis of patients with ATB across a large, diverse list of publicly available gene expression datasets, and evaluate their performance against the WHO/FIND TPPs.  Methods and Findings: We searched PubMed and Gene Expression Omnibus (GEO) in June 2018. We included all studies irrespective of study design and enrollment criteria. We found 16 gene signatures for the diagnosis of ATB compared to other clinical conditions in PubMed. For each signature, we implemented a classification model as described in the corresponding original publication of the signature. We identified 24 datasets containing 3,083 transcriptome profiles from whole blood or PBMC samples of healthy controls or patients with ATB, LTBI or other diseases from 14 countries in GEO. Using these datasets, we calculated weighted mean area under the receiver operating characteristic curve (AUROC), specificity at 90% sensitivity, specificity, and NPV for each gene signature across all datasets. We also compared diagnostic odds ratio (DOR), heterogeneity in DOR, and false positive rate (FPR) for each signature using bivariate meta-analysis.  Across 9 datasets of patients with culture-confirmed diagnosis of ATB, 11 signatures had weighted AUROC >0.8 and 2 signatures had weighted AUROC <0.6. All but two signatures had high NPV (>98% at 2% prevalence). Two gene signatures achieved the minimal WHO TPP for a non-sputum-based triage test. When including datasets with clinical diagnosis of ATB, there was minimal reduction in the weighted mean AUROC and specificity of all, but 3 signatures compared to when using only culture-confirmed data. Only 4 signatures had homogeneous DOR and lower FPR irrespective of how ATB was diagnosed; other signatures either had heterogeneous DOR or higher FPR or both. Finally, 7 of 16 gene signatures predicted progression from LTBI to ATB 6 months prior to sputum conversion with PPV > 6% at 2% prevalence. Our analyses may have under- or over-estimated performance of certain ATB diagnostic signatures because our implementation may be different from the published models for those signatures. We re-implemented published models because the exact models were not publicly-available.  Conclusions: We found that host-response based diagnostics could accurately identify patients with ATB and predict individuals with high risk of progression from LTBI to ATB prior to sputum conversion. We found that higher number of genes in a signature did not increase the accuracy of the signature. Overall, the Sweeney3 signature performed robustly across all comparisons. Our results provide a strong evidence for the potential of host response-based diagnostics in achieving the WHO goal of ending TB by 2035, and should be pursued for clinical implementation. | Abstract |
| **INTRODUCTION** | | |  |
| Rationale | 3 | Describe the rationale for the review in the context of what is already known. |  |
|  |  | Proliferation of host response-based transcriptome signatures despite the challenges described above raises several questions. First, do these signatures perform similar to each other in different clinical contexts in different patient populations? If yes, the second question is, do one or more of these signatures have potential to move towards translation into clinical practice cost effectively? Third, an overarching question is, does host response to *Mtb* has the potential to achieve the generalizability required to be used as a non-sputum-based triage test that meet TPPs described by the WHO and other groups for ending TB by 2035? | Introduction, paragraphs 1, 2, 3, and 4 |
| Objectives | 4 | Provide an explicit statement of questions being addressed with reference to participants, interventions, comparisons, outcomes, and study design (PICOS). |  |
|  |  | The focus of this study is to directly compare published gene signatures for diagnosis of patients with ATB across a large, diverse list of publicly available gene expression datasets, and evaluate their performance against the WHO/FIND TPPs. Within this study, we intend to address the following questions:  Which previously published gene signature for the diagnosis of ATB demonstrates the highest performance across publicly available gene expression datasets?  Which previously published gene signature for the diagnosis of ATB against one of either healthy controls, individuals with latent tuberculosis infection, or individuals with other diseases, demonstrates the highest performance across in subsets of gene expression datasets?  How does the performance of each signature compare the to WHO/FIND target product profiles? | Introduction, Paragraph 5 and 6 |
| **METHODS** | | |  |
| Protocol and registration | 5 | Indicate if a review protocol exists, if and where it can be accessed (e.g., Web address), and, if available, provide registration information including registration number. |  |
|  |  | A review protocol was not used for this study. A detailed explanation of the models and datasets including in the study are giving in the methods section and in the Information Sources section below. | Methods, Paragraph 1 in subsection “Prospective Analysis Plan” |
| Eligibility criteria | 6 | Specify study characteristics (e.g., PICOS, length of follow-up) and report characteristics (e.g., years considered, language, publication status) used as criteria for eligibility, giving rationale. |  |
|  |  | All participant eligibility was determined by the original studies designs outlined in the original publications on each model compared herein. The requirements for study and dataset inclusion are described in the Information Sources section below. | Methods, Paragraph 1 under subsection “Transcriptome datasets used for comparisons” |
| Information sources | 7 | Describe all information sources (e.g., databases with dates of coverage, contact with study authors to identify additional studies) in the search and date last searched. |  |
|  |  | We accessed two databases to find publicly available datasets for the analysis performed herein. The repositories we searched are to our knowledge exhaustive, every publicly available gene expression dataset is required to have an accession number for either of these 2 repositories. We searched these repositories in June of 2018 and included all datasets measure gene expression from blood of patients with ATB and at least one other group of individuals. We did not exclude datasets based on collection date or sample number. We excluded datasets measured using qPCR because they did not have enough coverage to capture all genes across the 16 signatures evaluated herein. The search terms used were as follows: TB (tuberculosis) gene expression, TB (tuberculosis) microarray, TB (tuberculosis) blood microarray, TB (tuberculosis) RNAseq, TB (tuberculosis) blood RNAseq, TB (tuberculosis) peripheral blood mononuclear cells gene expression, TB (tuberculosis) peripheral blood mononuclear cells microarray, TB (tuberculosis) peripheral blood mononuclear cells RNAseq, and possibly other related terms.  We accessed one repository of publications for the identification of publications describing gene expression signatures for the diagnosis of ATB. We performed an extensive search of published transcriptomic signatures for the diagnosis of ATB compared to a variety of other clinical features including healthy controls, LTBI, and other diseases in June 2018. We searched the National Center for Biotechnology Information’s repository of publications (PubMed) in june of 2018 for all publications describing a transcriptional signature for the diagnosis of active TB. We included all blood based transcriptional signatures that were specifically designed to diagnose ATB. We did not exclude any studies because of study criteria or date. Search terms included the following: TB (tuberculosis) gene signature, TB (tuberculosis) transcriptional signature, TB (tuberculosis) diagnostic, and possibly other related terms. We identified 13 publications describing 16 transcriptomic signatures for the diagnosis of ATB (**Table 1)**^17-27^. We note that we only considered transcriptome signatures that are described for diagnosis of ATB compared to healthy controls and patients with LTBI or other diseases. For instance, we did not include 4-gene RISK4 signature by Suliman *et al.* or 16-gene correlates of risk signature by Zak *et al.* as both signatures are designed to predict progression from LTBI to ATB, and not designed to diagnose active TB specifically. | Methods under subsections “Transcriptome Signatures for Comparison” and “Transcriptome datasets used for comparing signatures” |
| Search | 8 | Present full electronic search strategy for at least one database, including any limits used, such that it could be repeated. |  |
|  |  | The electronic search strategies used herein were relatively simple. For the purpose of identifying studies describing gene signatures for the diagnosis of ATB, we employed the following electronic search strategy:  We performed an extensive search of published transcriptomic signatures for the diagnosis of ATB compared to a variety of other clinical features including healthy controls, LTBI, and other diseases in June 2018. We searched the National Center for Biotechnology Information’s repository of publications (PubMed) in june of 2018 for all publications describing a transcriptional signature for the diagnosis of active TB. We included all blood based transcriptional signatures that were specifically designed to diagnose ATB. We did not exclude any studies because of study criteria or date. Search terms included the following: TB (tuberculosis) gene signature, TB (tuberculosis) transcriptional signature, TB (tuberculosis) diagnostic, and possibly other related terms. | Methods under subsection “Transcriptome datasets used for comparing signatures” |
| Study selection | 9 | State the process for selecting studies (i.e., screening, eligibility, included in systematic review, and, if applicable, included in the meta-analysis). |  |
|  |  | The process for study selection is described in the Informational sources section above. | Methods under subsection “Transcriptome datasets used for comparing signatures” |
| Data collection process | 10 | Describe method of data extraction from reports (e.g., piloted forms, independently, in duplicate) and any processes for obtaining and confirming data from investigators. |  |
|  |  | All microarray data was extracted from NCBI using the R MetaIntegrator package for extracting microarray data directly from NCBI. RNA-seq data was downloaded from NCBI and organized to replicate the structure of the data imported through the MetaIntegrator. We did not acquire data directly from any investigators. The studies listed in Table 2 can be obtained directly from the NCBI GEO at the following links:  GSE19491: https://www.ncbi.nlm.nih.gov/geo/query/acc.cgi?acc=GSE19491  GSE28623: https://www.ncbi.nlm.nih.gov/geo/query/acc.cgi?acc=GSE28623  GSE29536: https://www.ncbi.nlm.nih.gov/geo/query/acc.cgi?acc=GSE29536  GSE34608: https://www.ncbi.nlm.nih.gov/geo/query/acc.cgi?acc=GSE34608  GSE37250: https://www.ncbi.nlm.nih.gov/geo/query/acc.cgi?acc=GSE37250  GSE39939: https://www.ncbi.nlm.nih.gov/geo/query/acc.cgi?acc=GSE39939  GSE39940: https://www.ncbi.nlm.nih.gov/geo/query/acc.cgi?acc=GSE39940  GSE41055: https://www.ncbi.nlm.nih.gov/geo/query/acc.cgi?acc=GSE41055  GSE42834: https://www.ncbi.nlm.nih.gov/geo/query/acc.cgi?acc=GSE42834  GSE50834: https://www.ncbi.nlm.nih.gov/geo/query/acc.cgi?acc=GSE50834  GSE56153: https://www.ncbi.nlm.nih.gov/geo/query/acc.cgi?acc=GSE56153  GSE54992: https://www.ncbi.nlm.nih.gov/geo/query/acc.cgi?acc=GSE54992  GSE62147: https://www.ncbi.nlm.nih.gov/geo/query/acc.cgi?acc=GSE62147  GSE62525: https://www.ncbi.nlm.nih.gov/geo/query/acc.cgi?acc=GSE62525  GSE69581: https://www.ncbi.nlm.nih.gov/geo/query/acc.cgi?acc=GSE69581  GSE73408: https://www.ncbi.nlm.nih.gov/geo/query/acc.cgi?acc=GSE73408  GSE79362: https://www.ncbi.nlm.nih.gov/geo/query/acc.cgi?acc=GSE79362  GSE81746: https://www.ncbi.nlm.nih.gov/geo/query/acc.cgi?acc=GSE81746  GSE83456: https://www.ncbi.nlm.nih.gov/geo/query/acc.cgi?acc=GSE83456  GSE83892: https://www.ncbi.nlm.nih.gov/geo/query/acc.cgi?acc=GSE83892  GSE84076: https://www.ncbi.nlm.nih.gov/geo/query/acc.cgi?acc=GSE84076  GSE101705: https://www.ncbi.nlm.nih.gov/geo/query/acc.cgi?acc=GSE101705  GSE107731: https://www.ncbi.nlm.nih.gov/geo/query/acc.cgi?acc=GSE107731  GSE107994: https://www.ncbi.nlm.nih.gov/geo/query/acc.cgi?acc=GSE107994 | Methods under subsection “Transcriptome datasets used for comparing signatures” and in section “Data Availability” |
| Data items | 11 | List and define all variables for which data were sought (e.g., PICOS, funding sources) and any assumptions and simplifications made. |  |
|  |  | For the purpose of analysis within this study we sought the following information regarding participants, interventions, comparisons, outcomes, and study design:   - Studies that included individuals with active tuberculosis infect and other individuals with other diseases, individuals with latent tuberculosis infection, or healthy controls. - Studies that collected samples from patients with active tuberculosis prior to treatment on the infection with antibiotics. - No criteria for participants, outcomes, or further study design was used select or exclude studies. | Methods under subsections “Transcriptome Signatures for Comparison” and “Transcriptome datasets used for comparing signatures” |
| Risk of bias in individual studies | 12 | Describe methods used for assessing risk of bias of individual studies (including specification of whether this was done at the study or outcome level), and how this information is to be used in any data synthesis. |  |
|  |  | The explicit stated purpose of our analysis was to assess various host response-based gene signatures for diagnosis of active TB despite the heterogeneity present in real-world patient population. Therefore, we did not assess bias in individual studies. Instead, we assessed heterogeneity in diagnostic odds ratio for each signature to identify signatures that performed accurately and did not have heterogeneity between studies using bivariate meta-analysis. | Methods, in Prospective Analysis Plan. |
| Summary measures | 13 | State the principal summary measures (e.g., risk ratio, difference in means). |  |
|  |  | The principal summary measures reported in this study are AUROC, NPV, PPV, sensitivity, specificity, false positive rate, heterogeneity, and diagnostic odds ratio. | Methods, in Prospective Analysis Plan |
| Synthesis of results | 14 | Describe the methods of handling data and combining results of studies, if done, including measures of consistency (e.g., I^2^) for each meta-analysis. |  |
|  |  | The explicit stated purpose of our analysis was to assess various host response-based gene signatures for diagnosis of active TB despite the heterogeneity present in real-world patient population. Therefore, we did not assess bias in individual studies. Instead, we assessed heterogeneity in diagnostic odds ratio for each signature to identify signatures that performed accurately and did not have heterogeneity between studies using bivariate meta-analysis. | Methods, in Prospective Analysis Plan |

Page 1 of 2

| **Section/topic** | **#** | **Checklist item** | **Reported on page #** |
| --- | --- | --- | --- |
| Risk of bias across studies | 15 | Specify any assessment of risk of bias that may affect the cumulative evidence (e.g., publication bias, selective reporting within studies). |  |
|  |  | We did not perform assessment of risk of bias across studies. |  |
| Additional analyses | 16 | Describe methods of additional analyses (e.g., sensitivity or subgroup analyses, meta-regression), if done, indicating which were pre-specified. |  |
|  |  | We did not have a prospective analysis plan, except a broadly defined plan to compare published gene signatures for diagnosis of ATB using publicly-available gene expression data sets. | Methods, subsection Prospective analysis plan |
| **RESULTS** | | |  |
| Study selection | 17 | Give numbers of studies screened, assessed for eligibility, and included in the review, with reasons for exclusions at each stage, ideally with a flow diagram. |  |
|  |  | We included all publicly available datasets that included patients with active TB and at least one other group of individuals. We included are gene signatures that were derived for the purpose of diagnosing active TB against another group of individuals. No studies meeting these criteria were excluded to our knowledge regardless 0of other differences. | Results, paragraph 1 |
| Study characteristics | 18 | For each study, present characteristics for which data were extracted (e.g., study size, PICOS, follow-up period) and provide the citations. |  |
|  |  | For each dataset we present the following characteristics: accession number of the study, platform used to measure the gene expression, country where study was approved, tissue used in study, age range of participants (years), if HIV status was collected, if positive culture was used to confirm TB, number healthy controls, number of latently infected individuals, number of patients with ATB, number of patients with other diseases, total number of samples in the study.  For each model study included we present the following characteristics: first author’s name, PMID, discovery dataset GSE, indication of the diagnostic model, number of genes in the signature, type of model. | Methods, subsection “Transcriptome datasets used for comparing signatures” and Table 2 |
| Risk of bias within studies | 19 | Present data on risk of bias of each study and, if available, any outcome level assessment (see item 12). |  |
|  |  | The explicit stated purpose of our analysis was to assess various host response-based gene signatures for diagnosis of active TB despite the heterogeneity present in real-world patient population. Therefore, we did not assess bias in individual studies, but assessed heterogeneity in diagnostic odds ratio for each signature to identify signatures that performed accurately and did not have heterogeneity between studies. | Results, subsection “Comparison of accuracy for diagnosis of ATB using bivariate meta-analysis” |
| Results of individual studies | 20 | For all outcomes considered (benefits or harms), present, for each study: (a) simple summary data for each intervention group (b) effect estimates and confidence intervals, ideally with a forest plot. |  |
|  |  | There were no data included in this study for intervention. |  |
| Synthesis of results | 21 | Present results of each meta-analysis done, including confidence intervals and measures of consistency. |  |
|  |  | We report summary statistics calculated across multiple datasets within individual signatures. | Results, Tables 3, 4, and 5; subsections (1) Comparison of accuracy across only culture positive datasets for diagnosis of ATB, (2) Comparison of accuracy across all datasets for diagnosis of ATB, (3) Comparison of accuracy for diagnosis of ATB using bivariate meta-analysis, and (4) Signature performance predicting progression six months prior to active TB diagnosis. |
| Risk of bias across studies | 22 | Present results of any assessment of risk of bias across studies (see Item 15). |  |
|  |  | We estimated heterogeneity in diagnostic odds ratio across independent studies for each of the 16 gene signatures. | Results, subsection Comparison of accuracy for diagnosis of ATB using bivariate meta-analysis; Table 4 |
| Additional analysis | 23 | Give results of additional analyses, if done (e.g., sensitivity or subgroup analyses, meta-regression [see Item 16]). |  |
|  |  | No additional subgroup analyses were performed. |  |
| **DISCUSSION** | | |  |
| Summary of evidence | 24 | Summarize the main findings including the strength of evidence for each main outcome; consider their relevance to key groups (e.g., healthcare providers, users, and policy makers). |  |
|  |  | Across these biologically and technologically heterogeneous data, our comparison found that several gene signatures distinguished patients with ATB with moderate to high accuracy, although almost all signatures included a large number of genes that severely restrict their ability for cost-effective translation to clinical practice. Importantly, our analysis found that higher number of genes in a signature did not translate into higher accuracy across biologically heterogeneous data. Only 2 gene signatures (Sweeney3 and Sambarey10) satisfied the WHO TPP for a non-sputum-based triage test to identify which patients need further testing for confirming ATB, when comparing these signatures using datasets from only patients with culture-confirmed diagnosis of ATB.  When we included additional datasets that diagnosed ATB using other means (e.g., sputum microscopy), the accuracy of each of the 16 signatures reduced such that no signature satisfied the WHO TPP for the non-sputum-based triage test. The reduction in AUROC and specificity of Sweeney3 was minimal (AUROC=0.85, specificity=66%, sensitivity=90%). In contrast, Sambarey10 had substantial reduction in specificity that reduced by 15%. It is possible that inclusion of patients with ATB that are not diagnoses using positive culture caused the reduction in accuracy, which may underestimate the accuracy of these signatures. | Discussion, Paragraph 1 to 8 |
| Limitations | 25 | Discuss limitations at study and outcome level (e.g., risk of bias), and at review-level (e.g., incomplete retrieval of identified research, reporting bias). |  |
|  |  | Our analysis has a few limitations. First, we did not have access to the exact published models, or any hyper-parameters used to build the models for some of the gene signatures compared here. Therefore, we reimplemented those models to the best of our ability by following the details in the corresponding manuscripts. Hence, in the process of trying to build models that reproduce as close results as reported in the corresponding manuscript, the choices we made and hyper-parameters we inferred may be different from the original authors. This could result in overfitting, which in turn may result in reduced generalizability of a model in independent cohorts and underestimations of its accuracy. We recommend that when diagnostics signatures are published, the corresponding models should be made available along with a list of hyper-parameters and coefficients to enable reproducibility and comparison between models. Second, for some studies we were not able to use the original training data as they were not available. We chose another dataset that was similar to the discovery cohort described in a corresponding manuscript. This choice again may result in underestimation of accuracy. Therefore, if a model is extremely sensitive to training data, overfitting may have happened. This limitation points to the need for sharing underlying data used for building a classification model. Third, none of the datasets used in our analysis included patients with nontuberculous mycobacteria (NTM) infections. Therefore, it is not possible to evaluate whether the signatures compared here can differentiate patients with NTM infections or ATB. Fourth, patients with LTBI were defined using either TST or IGRA that could have different implications for progression to ATB. It is possible that the transcriptome profiles of patients using different diagnostic criteria for LTBI could have different transcriptome profiles. Our results showed that a few gene signatures demonstrated consistently high accuracy across datasets irrespective of how LTBI is defined, suggesting that host response to ATB is sufficiently different and robust to overcome the heterogeneity in clinical practice of how LTBI is defined. Importantly, our work described here points to future studies in how existing data could be used to identify differences in transcriptomes of patients with LTBI diagnosed with TST or IGRA. | Discussion, Paragraph 9 |
| Conclusions | 26 | Provide a general interpretation of the results in the context of other evidence, and implications for future research. |  |
|  |  | We found that when using datasets with only culture-positive diagnosis of ATB, only 2 signatures met the minimal WHO TPP for a non-sputum-based triage test. No signature met the minimal WHO TPP when datasets with clinical diagnoses of ATB were included, which may be due to lower accuracy of clinical diagnoses. Bivariate meta-analysis of these signatures further showed that only 4 out of 16 gene signatures had no heterogeneity irrespective of which datasets were included in the analysis. Further, we found 7 signatures met the TPP for a test for predicting progression from LTBI to ATB. Overall, across all comparisons, only Sweeney3 signature had less than 10 genes, met the WHO and FIND TPPs for non-sputum-based triage test for diagnosis of ATB and predicting progression from LTBI to ATB, and performed robustly with high DOR without heterogeneity and lowest FPR. We found that higher number of genes in a signature did not increase the accuracy of the signature. Our results provide a strong evidence for the potential of host response-based diagnostics in achieving the WHO goal of ending TB by 2035, and should be pursued for clinical implementation. | Discussion, under subsection “Conclusion” |
| **FUNDING** | | |  |
| Funding | 27 | Describe sources of funding for the systematic review and other support (e.g., supply of data); role of funders for the systematic review. |  |
|  |  | P.K. is supported in part by the Bill & Melinda Gates Foundation (OPT1113682) and National Institute for Allergy and Infectious Diseases grants R01 AI125197-01, U19AI109662, and U19AI057229. |  |

*From:*  Moher D, Liberati A, Tetzlaff J, Altman DG, The PRISMA Group (2009). Preferred Reporting Items for Systematic Reviews and Meta-Analyses: The PRISMA Statement. PLoS Med 6(6): e1000097. doi:10.1371/journal.pmed1000097

For more information, visit: **www.prisma-statement.org**.

Page 2 of 2
