# Supplementary material for: Host-response-based gene signatures for tuberculosis diagnosis: A systematic comparison of 16 signatures
Source: PLoS Med. 2019 Apr 23;16(4):e1002786. doi: 10.1371/journal.pmed.1002786 (PMC6478271; doi:10.1371/journal.pmed.1002786)
Supplement: S1 Text — (DOCX) [file pmed.1002786.s003.docx]

A systematic comparison of host response-based transcriptome signatures for diagnosis of tuberculosis

Hayley Warsinske^1,2^, Rohit Vashisht^1,2^, Purvesh Khatri^1,2,*^

^1^Institute for Immunity, Transplantation, and Infection, Stanford University, Stanford, CA 94305, USA

^2^Center for Biomedical Informatics, Department of Medicine, Stanford University, Stanford, CA 94305, USA

*Corresponding author: Purvesh Khatri ([pkhatri@stanford.edu](mailto:pkhatri@stanford.edu))

**Supplemental Methods**

*Models for each signature included in the study*

For each signature evaluated in this study a model is used to classify individuals as patients with ATB or other clinical conditions. To the best of our ability we reconstructed models corresponding to each signature as they we constructed in their original publications. At times we had to make minor assumptions or use a different training dataset, however these minor changes should not result in a misrepresentation of the signature. If such minor alterations are responsible for major differences in signature outcome, it speaks to the irreproducibility of the work and should be identified in the original publication. For most calculations of prediction score (unless otherwise specified) log2 normalized expression values of gene expression data were used. The majority of signatures included in this study were derived from and tested on log2 normalized data. Any instance otherwise is specifically addressed.

*Random Forest Models*

Four signatures, daCosta3, Maertzdorf4, Maertzdorf15, and Verhagan10, were evaluated using random forest models. Random forest (RF) models were constructed using the randomForest function from the R package randomForest (ref). For each signature, the expression of signature genes and associated diagnosis for samples in the training data was used to train a random forest model. We grew 5000 trees to train each model. A example of the function and inputs is as follows:

Random forest model <- randomForest(x = signature genes expression, y = diagnosis, ntree = 5000)

Further application of random forest models was performed using the predict function from the R package stats (ref). Prediction scores were used to calculate AUROC and other statistics. An example of the function and inputs is as follows:

Prediction score <- predict(object = model, newdata = signature genes expression, type = "prob")

The RF models associated with the Maertzdorf4 and Maertzdorf15 signatures were originally trained on data that was normalized by median and interquartile range. Because this normalization was inter-dataset we felt that is was acceptable to perform this normalization for data as it was evaluated by the Maertzdorf4 and Maertzdorf15 signatures. We used the normalize function from the R package BBmisc (ref) to perform this normalization across samples within a dataset. It is unlikely that this normalization step have substantial influence on the prediction scores from the Maertzdorf4 and Maertzdorf15 RF models, however, in an effort to accurately recreate the original models we choose to perform this step.

The RF model originally published with the daCosta3 signature was trained on quantitative PCR data that was not made publicly available. Because we did not have access to the original training data for this model we chose a different but suitable dataset for training. In the original publication, the daCosta3 signature was tested on the dataset GSE42834. Because this dataset was used in the original publication, we deemed it an appropriate substitution for the training data we did not have access to.

*Linear Discriminatory Analysis Models*

Two signatures, Jacobsen3 and Sambarey10, were evaluated using linear discriminatory analysis (LDA) functions. LDA models were constructed using the lda function from the R package MASS (ref). For each signature an LDA model was trained based on the expression of signature genes and the known diagnosis of samples in the training dataset. For the Jacobsen3 and Sambarey10 signatures, models was constructed respective as follows:

Jacobsen3 model <- lda(Diagnosis ~ RAB33A + FCGR1A + LTF, training dataset)

Sambarey10 model <- lda(Diagnosis ~ FCGR1A + HK3 + RAB13 + RBBP8 + IFI44L + TIMM10 + BCL6 + SMARCD3 + CYP4F3 + SLPI, training dataset)

Further application of LDA models was performed using the predict function from the R package stats (ref). Prediction scores were made and the linear discriminant was used to calculate AUROC and other statistics (see example of predict function and inputs in random forest).

The LDA model associated with the Jacobsen3 signature in its original publication was trained on GSE6112. Although we did have access to the data associated with the original model, the phenotypic descriptions of each sample were unclear limiting our ability to use the data in any capacity. We therefor choose a different, suitable dataset to train the Jacobsen3 LDA model. We choose to train the model on GSE19491 for several reasons. GSE19491 was the most commonly used training dataset across the studies included in this analysis and thus we felt is should not introduce any strong artifacts. Because GSE6112 compares patients with ATB to individuals with LTBI, we excluded any heathy controls and patients with other diseases from GSE19491 when training the Jacobsen3 LDA model.

*Ridge Logistic Regression*

One signature, Leong24, was evaluated using a rigid logistic regression (RLR) model. The RLR model was constructed using the cv.glmnet function from the glmnet R package (ref) with default parameters as described in the original publication of the signature (ref). The model was constructed as shown in the following example:

Leong24 model <- cv.glmnet(x = expression of signature genes in training data, y = diagnosis of training data)

Further application of the Leong24 model was performed using the predict function from the R package stats (ref). Prediction scores were used to calculate AUROC and other statistics. An example of the prediction function and inputs is as follows:

Prediction score <- predict(Leong24 Model$glmnet.fit, s = opt_lambda, newx = signature gene expression , na.action = na.omit)

*Support Vector Machines*

Three signatures, Bloom144, Suliman4, and Zak16, were evaluated using support vector machines (SVM). SVM models were constructed using the svm function from the e1071 package in R (ref). For the Bloom14 and Zak16 signatures, an SVM model was trained on the expression of signature genes and sample diagnosis in the training dataset as follows:

Support vector machines <- svm(x = signature gene expression, y = diagnosis, type = "nu-regression", kernel = "linear", cost = 100, cachesize = 5000, tolerance = 0.01, shrinking = FALSE, cross = 3)

Further application of the Bloom14 and Zak16 SVMs was performed using the predict function from the R package stats (ref). Prediction scores were used to calculate AUROC and other statistics. (see example of predict function and inputs in random forest).

The Suliman4 signature required an extra data process step before constructing and applying the SVM. The Suliman4 signature uses paired ratios of gene expression rather than gene expression values as inputs for the SVM. Paired ratios of gene expression are calculated by taking the expression value of a gene upregulated in disease and dividing it my the expression value for a gene that is downregulated in disease. This process is repeated for each possible pair of up and downregulated genes. In the Suliman4 signature GAS6 and SEPT4 are upregulated while CD1C and BLK are down regulated in disease compared to healthy. Thus, the paired ratios are GAS6/CD1C, SEPT4/BLK, SEPT4/CD1C, GAS6/BLK. The Suliman4 SVM was trained on the paired ratios of signature genes and sample diagnosis in the training dataset as follows:

Support vector machines <- svm(x = paired ratios, y = diagnosis, type = "nu-regression", kernel = "linear", cost = 100, cachesize = 5000, tolerance = 0.01, shrinking = FALSE, cross = 3)

Paired ratios were calculated for all datasets before further application of the Suliman4 SVM. The predict function from the R package stats (ref) was used to generate prediction scores. Prediction scores were used to calculate AUROC and other statistics. (see example of predict function and inputs in random forest).

*K-Nearest Neighbors Models*

Two signatures, Berry86 and Berry393, use K-nearest neighbors models to classify individuals with active TB or other clinical status. In the original publication of the Berry signatures all data (training and testing) was co-normalized. This is an important step for the efficacy of K-nearest neighbors models, however is not a very clinically relevant practice. In this paper we chose not to co-normalize any of the data. We acknowledge that by not co-normalizing the data K-nearest neighbors models are disadvantaged. However, because the only signatures affected by this decision have higher than clinically-relevant gene counts, we did not feel that the overall results of the paper were altered by this choice. We applied the K-nearest neighbors models to each independent dataset as was done for all models. The results thereby reflect how K-nearest neighbors would perform across heterogenous data in clinical practice. The knn function from the class package in R (ref) was used to generate prediction scores from the according the following example:

Prediction score <- knn(train = training data signature gene expression, test = testing data signature gene expression, cl= training data diagnosis, k=10, prob=T)

*Difference of Sums*

Two signatures, Anderson42 and Anderson51, use difference of sums to calculate prediction scores. Difference of sums is calculated by subtracting the sum of the expression of genes downregulated in disease from the sum of the expression of genes upregulated in disease producing a prediction score. An example of the difference of sums calculation is as follows:

Prediction score <- sum(upregulated gene 1, upregulated gene 2, … upregulated gene n) - sum(downregulated gene 1, downregulated gene 2, … downregulated gene n)

The difference of sums is a simple arithmetic calculation and can be applied generally once discovery of genes has been completed. Discovery data is use to identify the genes in the gene signatures but otherwise a prediction score from the difference of sums can be calculated for any dataset so long and the gene in the equation are known.

*Difference of Arithmetic Means*

Three signatures in this analysis, Kaforou27, Kaforou44, and Kaforou52, use difference of arithmetic means to calculate prediction scores. Similar to the difference of sums explained above, difference of arithmetic means is calculated by subtracting the mean of the expression of genes downregulated in disease from the mean of the expression of genes upregulated in disease producing a prediction score. Difference of arithmetic means specifies that the gene expression values are not log normalized. An example of the difference of sums calculation is as follows:

Prediction score <- mean(upregulated gene 1, upregulated gene 2, … upregulated gene n) - mean(downregulated gene 1, downregulated gene 2, … downregulated gene n)

*Difference of Geometric Means*

One signature, Sweeney3, uses difference of geometric mean to calculate prediction score. Similar to difference of arithmetic means, difference of geometric means is calculated by subtracting the mean of the expression of genes downregulated in disease from the mean of the expression of genes upregulated in disease producing a prediction score. However, in order to calculate geometric mean, the means are taken of log2 normalized gene expression values.
